# Ginsenoside in the treatment of type 2 diabetes and its complications: a promising traditional chinese medicine

**DOI:** 10.3389/fphar.2025.1593780

**Published:** 2025-05-13

**Authors:** Yingying Liu, Yang Ju, Yanjun Wang, Xiaoyan Cui, Yunwei Sun, Ping Hu, Yan Chen

**Affiliations:** ^1^ Department of Endocrinology, The Second Hospital of Jilin University, Changchun, China; ^2^ Department of Otolaryngology - Head and Neck Surgery, The Second Hospital of Jilin University, Changchun, China

**Keywords:** type 2 diabetes mellitus, ginsenoside, traditional Chinese medicine, therapeutic affect, complications

## Abstract

Type 2 diabetes mellitus (T2DM), a chronic condition commonly observed in adults, particularly among the elderly, is characterized by a dysfunctional insulin response that impairs blood glucose regulation, resulting in persistent hyperglycemia. Ginseng, a medicinal plant with significant economic value and a longstanding history of therapeutic use in Asia, has shown efficacy against various diseases. Extensive clinical and experimental studies highlight ginsenosides, its primary bioactive compounds, for their multiple therapeutic effects across a range of conditions, including endocrine, cardiovascular, and central nervous system disorders. Various ginsenoside types have demonstrated potential in lowering blood glucose levels, reducing insulin resistance, and alleviating complications through the modulation of key protein targets and signaling pathways. This review consolidates the pharmacological actions and mechanisms of distinct ginsenosides in managing diabetes and its complications, offering a theoretical foundation for further pharmacological research and novel drug development for T2DM treatment, while also providing robust theoretical support for future clinical applications.

## 1 Introduction

Diabetes mellitus (DM) is a metabolic disorder characterized by hyperglycemia arising from inadequate insulin secretion and/or compromised insulin efficacy (Wang and Cao, 2025). The International Diabetes Federation (IDF) estimates that DM currently impacts approximately 536.6 million adults globally, with projections indicating an increase to 783.2 million by 2045 (Yang et al., 2024). The rising prevalence of DM, particularly type 2 diabetes mellitus (T2DM), stems from a complex interplay of genetic predispositions—such as obesity, impaired postprandial insulin release, and damage to certain pancreatic β cells—and environmental factors, including obesity, poor dietary habits, physical inactivity, and aging (Młynarska et al., 2025). This results in sustained hyperglycemia and reduced insulin sensitivity, which cumulatively contribute to various metabolic disorders.

**FIGURE 1:**
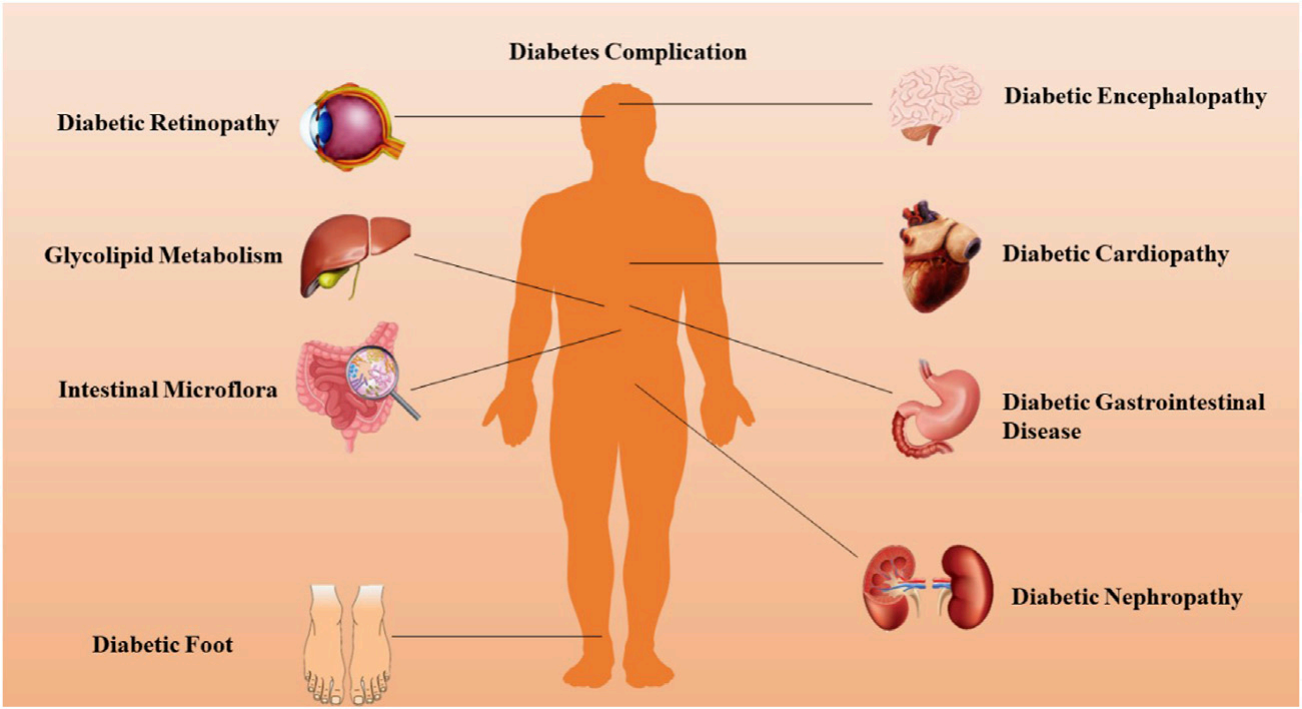
DM can lead to complications in a variety of human systems or organs.

T2DM significantly disrupts numerous organ systems by inducing profound alterations across nearly all cellular metabolic pathways. Chronic complications of DM often arise from either insulin deficiency or resistance, accompanied by persistent hyperglycemia, dyslipidemia, and other metabolic irregularities (Bilal and Pratley, 2025). These complications encompass macrovascular diseases, notably cardiovascular and cerebrovascular conditions primarily manifesting as atherosclerosis, and microvascular diseases, which involve thickening of the basement membrane and deposition of transparent material in tissues such as the retina, kidneys, and nerves. Key examples include diabetic retinopathy (DR), diabetic nephropathy (DN), and neuropathy (Alexander et al., 2024). Emerging evidence suggests that this binary classification of DM complications may require refinement, as many complications resist categorization into solely microvascular or macrovascular types. Instead, chronic DM complications can be grouped into pathological categories involving vascular, parenchymal, and mixed tissue, as illustrated in Figure 1. These complications constitute the primary causes of morbidity and mortality associated with DM, imposing a growing strain on healthcare systems worldwide, affecting both developed and developing nations (Singh et al., 2025).

Traditional diabetes therapies, including thiazolidinediones (SU), biguanides (BG), and α-glucosidase inhibitors, frequently carry significant side effects such as hypoglycemia, drug resistance, edema, and weight gain (Zhang et al., 2025). With advancements in diabetes research, therapeutic approaches have shifted from merely enhancing insulin’s hypoglycemic effects to broader strategies that regulate glucose metabolism, increase insulin receptor sensitivity, inhibit insulin resistance, control non-enzymatic glycosylation of proteins, and decrease fatty acid metabolism (Shahcheraghi et al., 2021). Current diabetes management primarily emphasizes insulin or peptide derivatives, oral antidiabetic drugs, and dietary modifications. However, daily intravenous insulin or peptide injections are inconvenient and burdensome for patients (Scairati et al., 2025), while long-term oral administration of chemical drugs poses risks of toxicity. Despite numerous strategies and medications developed for diabetes prevention and treatment, the outcomes remain largely suboptimal. Consequently, there is an urgent demand for new, effective, and safer natural hypoglycemic agents as alternative therapies for diabetes and its complications. Recent years have witnessed a growing interest in traditional Chinese medicine for diabetes treatment, particularly ginseng.

Ginseng, a perennial herb reaching up to 60 cm in height, is primarily cultivated in northeastern China, Korea, North Korea, and Japan (Iqbal et al., 2025). The ginseng shares a basic structure with steroid hormones, containing saponins, polysaccharides, polyacetylene, phenols, and alkaloids, each with a rigid tetracyclic steroid backbone of 17 carbon atoms (Li et al., 2024). Ginsenosides, a principal class of natural triterpene saponins within ginseng, are recognized as key contributors to its antidiabetic properties. To date, nearly 200 ginsenosides have been identified in ginseng plants and heat-processed ginseng products (Qi et al., 2022). Ginsenosides are generally categorized into two subtypes: protopanaxadiol (PPD) and protopanaxatriol (PPT). PPD-type ginsenosides include Rb1, Rb2, Rb3, Rc, Rd, Rh2, Rg3, and F2, while PPT-type ginsenosides encompass Re, Rf, Rg1, Rg2, and Rh1 (Liu et al., 2024). These compounds hold therapeutic potential for treating a wide range of conditions, including diabetes, cancer, digestive diseases, cardiovascular diseases and nervous system disorders (Niu et al., 2025).

Increasing evidence from cell, animal, and clinical studies demonstrates that various ginsenosides exert antidiabetic effects through multiple mechanisms. However, systematic evaluations detailing the specific antidiabetic mechanisms and the preventive effects of different ginsenoside types on diabetes complications remain lacking. This review compiles and analyzes recent studies (from 2020 onward) on ginsenosides in the treatment of diabetes and its complications across different systems or organs, providing a comprehensive theoretical foundation for their application in diabetes management.

## 2 Specific varieties of ginsenoside ingredients

The active ingredients associated with the ginsenosides used in the treatment of type 2 diabetes and its complications are shown in Table 1.

**TABLE 1:** Detailed information on the kinds of beneficial ginsenoside properties in the treatment of type 2 diabetes and its complications with different systems.

Ginsenoside	Mechanism	Signal path or receptor	References
Cardiovascular system			
Rb1	• anti-oxidative stress	AMPK//Nrf2/HO-1	Bingbing et al. (2023)
Rb1	• decrease in extracellular Ca^2+^ influx		Park et al. (2023)
Rb1	• lower lipid levels • attenuate oxidative stress, hypertrophy, inflammation, fibrosis, and apoptosis in cardiomyocytes	adipocytokine pathway	Zhang et al. (2022)
Rb1	• ameliorate endothelial cell injury and atherosclerosis		Wang et al. (2022a)
Rb1	• anti-oxidative stress • reduce dysfunction of RyR2	RyR2	Feng et al. (2024)
Rb1	• alleviate myocardial lipid accumulation • alleviate mitochondrial injury • attenuate ventricular diastolic dysfunction	Mfn2	Ji et al. (2024)
Rb1	• alleviate collagen deposition and degradation	AMPK	Zhang et al. (2021a)
Rb1	• improve cardiac dysfunction and abnormal cardiomyocytes calcium signaling	O-GlcNA	Qin et al. (2019a)
Rb1	• anti-apoptosis	PI3K/AKT	Wu et al. (2011)
Rg1	• alleviate the development of mitochondrial dysfunction and oxidative stress	calpain-1/ROS/PKC-β	Lu et al. (2023)
Rg1	• induce macrophage M2 polarization	NOTCH	Zhen et al. (2024)
Rg1	• anti-apoptosis	HIF-1/α-ERK	Yuan et al. (2019)
Rg1	• anti-inflammation • anti-oxidative stress	AMPK/Nrf2/HO-1	Qin et al. (2019b)
Rg1	• reduce the cerebral infarction volume • promote neuronal recovery		Shen et al. (2017)
Rg1	• anti-apoptosis		Yu et al. (2016)
Rg1	• anti-apoptosis • anti-oxidative stress		Yu et al. (2015)
Rg1	• improve angiogenesis • anti-apoptosis		Yang et al. (2012)
Rg3	• inhibit vascular smooth muscle cell proliferation and migration	PPARγ	Guo et al. (2018a)
Rg3	• induce macrophage M2 polarization	PPARγ	Guo et al. (2018b)
Rg3	• improve adiponectin secretion • promote adiponectin signaling	PPARγ	Zhang et al. (2023)
Rk1	• ameliorate endothelial dysfunction • anti-oxidative stress	PPAR/eNOS	Miao et al. (2024)
Rh2	• improves cardiac fibrosis	PPARγ/STAT3	Lo et al. (2017)
Fc	• promote endothelial cell autophagy		Liu et al. (2019a)
Fc	• anti-apoptosis • anti-inflammation • promote proliferation	PPARγ	Liu et al. (2018)
Re	• anti-angiopathy	P38MAPK/ERK1/JNK	Shi et al. (2016)
Notoginsenoside R1 (NR1)	• promote viability inhibition • anti-apoptosis • enhance tube formation ability • inhibit oxidative stress and inflammatory	Mir-147a and MyD88/TRAF6/NF-κB	Li and Huang (2021)
NR1	• anti-apoptosis	miR-21 and PI3K/AKT	Liu et al. (2019b)
Korean Red Ginseng (KRG)	• antihyperglycemic and antioxidative effects		Hossain et al. (2020)
Digestive system			
Rb1	• modulate specific gut microbes and related metabolites		Wang et al. (2023)
Rb1	• enhance liver glycogen production	15-PGDM	Liang et al. (2023)
Rb1	• anti-inflammation • anti-apoptosis	Akt/FOXO1	Su et al. (2022a)
Rb1	• increase insulin sensitivity	11β-HSD1	Song et al. (2017)
Rb1	• stimulate GLP-1 secretion in enteroendocrine L cells	GLP-1	
Rb1	• stimulate glucose uptake	GLUT1/GLUT4	Shang et al. (2008)
Rb1	• attenuate insulin resistance		Xiong et al. (2010)
Rb2	• attenuate insulin resistance • reduces fat mass • improve insulin sensitivity	AKT	Dai et al. (2018)
Rb2	• inhibit gluconeogenesis	ER/AMPK	Lee et al. (2011a)
Rb3	• improve oral glucose tolerance • repaire injured pancreas tissues		Bu et al. (2012)
Rg1	• improve islet injury and tissue inflammation • raise serum insulin, and tissue autophagy marker		Zong et al. (2023)
Rg1	• anti-apoptosis	AMPK/mTOR	Chen et al. (2023)
Rg1	• increase the proportions of bacteria		Peng et al. (2022)
Rg1	• anti-inflammation • anti-pyroptosis • anti-oxidative stress	NLRP3 and Keap1/Nrf2/HO-1	Gao et al. (2020)
Rg1	• increase the uptake of glucose • decrease the output of glucose	AKT/GSK3β	Fan et al. (2019a)
Rg1	• anti-inflammation		Fan et al. (2019b)
Rg1	• inhibit hepatic gluconeogenesis	AKT	Liu et al. (2017)
Rg1	• suppress hepatic glucose production	LKB1/AMPK/FoxO1	Kim et al. (2010)
Rg1	• inhibit obesity • improve insulin resistance and glucose intolerance	AMPK	Li et al. (2018)
Rg1+Rb1	• anti-apoptosis	Fas	Chen et al. (2012)
Rb1+Rg1	• enhance secretion and viability	PKA	Park et al. (2008a)
Rk1	• anti-apoptosis	IGF-1R	Vong et al. (2024)
Rg2	• inhibit hepatic glucose production	SHP/GSK3β/AMP	Yuan et al. (2012)
Rg3	• hyperglycemia • insulin resistance therapy		Han et al. (2024)
Rg3	• anti-apoptosis • increase proliferation	INS-1/ERK/p38 MAPK	Kim et al. (2016)
Rg3	• stimulate GLP-1 secretion in enteroendocrine L cells	GLP-1	Kim et al. (2015)
Rg3	• anti-apoptosis		Kim et al. (2014)
Rg3	• enhance glucose-stimulated insulin secretion	AMPK	Park et al. (2008b)
Rg3+Re	• stimulate glucose uptake	IRS-1/PI3K	Lee et al. (2011b)
Rg5	• attenuate hepatic glucagon response	HIF-1α	Xiao et al. (2017)
Rg5	• improve insulin resistance • improve mitochondrial biogenesis	Sirt1/PGC-1α	Zhu et al. (2021)
Rg5	• reverse gut microbiota dysbiosis and diabetes-associated metabolic disorders		Wei et al. (2020)
Rd	• increase the diversity of gut microbiota, increased the abundance of beneficial bacteria		Wang et al. (2023)
Rd	• anti-apoptosis		Kaviani et al. (2019)
Re	• increase insulin resistance	AMPK	Quan et al. (2012)
Rh2	• anti-apoptosis	AKT/Foxo1/PDX-1	Wang et al. (2012)
Rh4	• improves pancreatic β-cells dysfunction • anti-oxidative stress	Nrf2	Liu et al. (2021)
Rk3	• mediate hepatic gluconeogenesis and lipid accumulation	AMPK/AKT	Liu et al. (2019c)
F4	• enhance insulin sensitivity • alleviate endoplasmic reticulum (ER) stress	IRE-1/TRAF2/JNK	Zhao et al. (2023)
CK	• inhibit the macrophage activation	PPAR γ/NF-κB	Xu et al. (2022)
CK	• modulate the abundance of L-cells	TGR5/YAP	Tian et al. (2022b)
CK	• remodel gut microbiota and bile acid metabolism	TGR5	Tian et al. (2022a)
CK	• anti-inflammation	RhoA/ROCKs/YAP	Tian et al. (2021)
CK	• suppress hepatic gluconeogenesis	AMPK	Wei et al. (2015)
CK	• increase insulin resistance	PI3K/AKT	Jiang et al. (2014)
CK	• anti-apoptosis	AMPK/JNK	Guan et al. (2014)
CK	• enhance insulin secretion	GLUT2	Gu et al. (2013)
CK	• suppress the hepatic gluconeogenesis		Li et al. (2012)
CK	• attenuate glucose intolerance and hepatic steatosis	AMPK	Hwang et al. (2018)
CK	• anti-apoptosis	SAPK/JNK	Kim et al. (2009a)
T19	• lower the levels of blood glucose and lipid • alleviate insulin resistance • improve histological pathology of liver and pancreas	AMPK/PI3K	Xu et al. (2020)
NR1	• alleviate apoptosis and dysfunction	miR-29a	Chen et al. (2019)
Red Ginseng	• improve lipid deposition		Huang et al. (2022)
Malonyl Ginsenoside (MGR)	• improve glucose and lipid metabolism and insulin resistance	IRS1/PI3K/AKT and MAPK	Wang et al. (2022b)
Nervous system			
Rb1	• anti-inflammation	Nrf2/NLRP3	Zhai et al. (2018)
Rb1	• improve glucose metabolism • ameliorate depression-like behavior		Zhang et al. (2021b)
Rb1	• improve cognitive ability • improve glucose tolerance	Cdk5/p35-NMDAR-IDE	Yang et al. (2020)
Rb1	• protect neurons	GSK3β/CHOP	Liu et al. (2014)
Rb1	• anti-oxidative stress and activation of the mitochondrial apoptosis		Li et al. (2017)
Rb1	• anti-oxidative stress • anti-apoptosis		Xue et al. (2012)
Rg1	• improve synaptic dysfunction • improve memory impairment and neuronal injury	PLC-CN-NFAT1	Dong et al. (2023)
Rg3	• prevent degeneration of neurons • exert the antioxidant effect		Liu et al. (2015)
Re	• ameliorate brain insulin resistance and cognitive dysfunction	JNK	Kim et al. (2017)
Re	• anti-inflammation		Liu et al. (2012)
NR1	• neuroprotective and neurotrophic function	miR-503	Wang et al. (2019b)
CK	• inhibit brain oxidative/nitrosative damage		Tian et al. (2018)
CK	• ameliorate glucose tolerance, insulin sensitivity, and dyslipidemia • suppress oxidative stress and inflammatory response	ER/NLRP3	Li et al. (2020)
Muscles			
Rg1	• promote glucose uptake	GLUT4	Lee et al. (2012)
Rg3	• promote myoblastic differentiation • protect mitochondrial function	AMPK/Smad	Wang et al. (2024a)
Rg3	• improve insulin signaling and glucose uptake	IRS-1/GLUT4	Kim et al. (2009b)
Rc	• stimulate glucose uptake	AMPK	Lee et al. (2010)
CK	• anti-apoptosis • anti-inflammation • anti-oxidative stress • lower metalloproteinase (MMP)	PPARγ	Cho et al. (2024)
CK	• induce mitophagy	DRP1/PINK1	Li et al. (2023)
Diabetic retinopathy			
Rb1	• anti-oxidative stress	Nrf2	Dong et al. (2019)
Rb1	• anti-oxidative stress	NAD/PARP/SIRT	Fan et al. (2019c)
Rg1	• prevent synaptic neurodegeneration	IRS-1/Akt/GSK3β	Ying et al. (2019)
Rg1	• inhibits mesenchymal activation and fibrosis	miR-2113/RP11-982M15.8/Zeb1	Xue et al. (2018)
Rg1	• inhibite cell proliferation, cell cycle progression, angiogenesis, and the production of inflammatory cytokines and growth factors	TLR4/NF-κB	Xue et al. (2023)
Rg3	• prevent neovascularization		Sun and Zhou (2010)
Re	• anti-apoptosis • anti-oxidative stress	PI3K/AKT and HIF-1α/VEGF	Xie et al. (2020)
Re	• anti-oxidant stress • anti-hyperlipidemic		Cho et al. (2006)
NR1	• improve retinal vascular degeneration • improve retinal thickness • improve retinal function	PINK1	Zhou et al. (2019)
NR1	• modulate the intracellular redox state		Fan et al. (2017)
Panax notoginseng saponins (PNS)	• anti-inflammation	NF-κB	Wang et al. (2024b)
Lung			
Rb1	• anti-oxidative damage and inflammatory infiltration		Su et al. (2022b)
Rg3	• anti-inflammation	PI3K/MAPK	Wang et al. (2019a)
Urinary system			
Rb1	• inhibit aldose reductase activity	AR	He et al. (2022)
Rg1	• improve lipid deposition, fibrosis, and ROS production	NOX4-MAPK	Ji et al. (2023)
Rg1	• ameliorate renal lipid accumulation, pathological damage, and glomerular fibrosis	TRPC6/NFAT2	Han et al. (2023)
Rg3	• induce mesangial cells proliferation • anti-apoptosis	miR-216a-5p/MAPK	Chen et al. (2024)
Rg3	• reduce inflammation and fibrosis	PPARγ	Sui et al. (2023)
Rg3	• improve anti-oxidative activity • reduce renal inflammation	MAPK/NF-κB	Li et al. (2021)
Rg3	• anti-inflammation		Zhou et al. (2020)
Rg3	• anti-oxidative stress		Kang et al. (2010)
Rg5	• anti-inflammation • anti-oxidative stress	NLRP3/MAPK	Zhu et al. (2020)
Rd	• antioxidative and antiapoptotic activities		Jung et al. (2021)
Rh1	• anti-inflammation • anti-apoptosis	AMPK/PI3K/AKT	Su et al. (2021)
NR1	• anti-inflammation • anti-apoptosis	PI3K/AKT/NF-κB	Huang et al. (2016)
NR1	• inhibit apoptosis and renal fibrosis • anti-oxidative stress	Nrf2/HO-1	Zhang et al. (2019)
CK	• anti-inflammation	NLRP3/NF-κB/p38	Song et al. (2018)
CK	• inhibit microbially produced imidazole propionate	TLR4	Chen et al. (2022)
CK	• enhance antioxidant capacity		Shao et al. (2015)
Notoginsenoside Fc	• endothelial cells pyroptosis • mitochondrial dysfunction	HMGCS2	Shen et al. (2024)
PNS	• anti-inflammation • anti-oxidative stress	SIRT1	Du et al. (2016)
Wound healing			
Rb1	• stimulate the wound-healing activity of fibroblasts		Namgoong et al. (2019)
Rg1	• promote cell proliferation, migration and angiogenesis • anti-apoptosis	miR-489-3p/SIRT1 and PI3K/AKT/eNOS	Huang et al. (2021)
Rg1	• promote angiogenesis	miR-23a/IRF-1	Cai et al. (2019)
Rg5	• fuel the efferocytosis of dendritic cells	NF-κB	Xia et al. (2023)
PPD	• stimulate angiogenesis	PI3K/Akt/mTOR and Raf/MEK/ERK	Zhang et al. (2017)

### 2.1 Cardiovascular system

DM is an important risk factor for cardiovascular diseases. Long-term hyperglycemia leads to vascular endothelial damage through oxidative stress, inflammatory response and metabolic disorders, and accelerates the process of atherosclerosis. Coronary atherosclerotic heart disease is often complicated in diabetic patients, which is manifested as angina pectoris and myocardial infarction. At the same time, myocardial microangiopathy can lead to diabetic cardiomyopathy, leading to ventricular remodeling and heart failure.

#### 2.1.1 Endothelial cell dysfunction

Under high glucose conditions, the polyol pathway is activated, and advanced glycation end products accumulate, ultimately triggering an oxidative stress response that damages vascular endothelial cells—an initiating and central factor in diabetes-associated vascular disease (Yang and Liu, 2022).

In response, numerous scholars have systematically investigated this critical mechanism. Findings indicate that Rb1 enhances arterial flexibility, aortic compliance, and endothelium-dependent vasodilation by inhibiting the transforming growth factor-β1 (TGFβ1)/Smad2/3 pathway (Zhang J. H. et al., 2021), reducing oxidative stress (Park et al., 2023), and preventing endothelial-mesenchymal transition (EndMT), apoptosis, and mitochondrial damage (Wang D. S. et al., 2022). Additionally, Rk1 activates the peroxisome proliferator-activated receptor (PPAR)/endothelial nitric oxide synthase (eNOS) pathway, which alleviates endothelial dysfunction and suppresses oxidative stress in diabetic vascular tissue (Miao et al., 2024). Rg1 protects against vascular endothelial dysfunction (VED) by inhibiting the calpain-1/reactive oxygen species (ROS)/protein kinase C-β (PKC-β) axis, thereby mitigating mitochondrial dysfunction and oxidative stress (Lu et al., 2023). At concentrations of 10–40 μM, NR1 downregulates the MyD88/tumor necrosis factor receptor-associated factor 6 (TRAF6)/nuclear factor kappa-B (NF-κB) pathway by upregulating miR-147a, which suppresses oxidative stress, inflammation, and apoptosis while enhancing tube formation (Li and Huang, 2021). Furthermore, a study on Korean Red Ginseng (KRG) demonstrated improved cardiac function in diabetic rats by normalizing ejection fraction, fractional shortening, and vascular reactivity, although the study was limited to animal models and did not extend to cell-level mechanistic analysis (Hossain et al., 2020).

#### 2.1.2 Diabetic cardiomyopathy (DCM)

Diabetic cardiomyopathy (DCM) is a distinct form of cardiomyopathy in patients with DM that is not attributable to hypertensive heart disease, coronary atherosclerosis, or other cardiac conditions. It results in pathological abnormalities, including cardiomyocyte apoptosis, left ventricular dysfunction, cardiac remodeling, inflammation, oxidative reactions, and myocardial metabolic disorders (Zhao et al., 2022). Feng et al. (2024) demonstrated that Rb1 improves myocardial injury in diabetic rats by reducing cardiomyocyte apoptosis and mitigating oxidative damage, although the specific mechanisms were not explored. Recent studies have expanded on this, indicating that Rb1 can not only lower lipid levels through adipocytokine-mediated pathways (Zhang et al., 2022) but also modulate the adenosine 5′-monophosphate-activated protein kinase (AMPK)/nuclear factor erythroid 2-related factor 2 (Nrf2)/heme oxygenase 1 (HO-1) signaling pathway (Bingbing et al., 2023). Furthermore, Rb1 has been shown to alleviate hyperglycemia/hyperlipidemia-induced ventricular diastolic dysfunction, metabolic disorders, oxidative stress, cardiomyocyte apoptosis, and fibrosis by inhibiting mitochondrial damage (Ji et al., 2024). Zhang et al. (2023) highlighted the therapeutic potential of Rg3 in DCM, noting that Rg3 counteracts lipid accumulation-induced dysfunction in cardiac tissue by enhancing adiponectin secretion and signaling. In addition, Lu et al. (Zhen et al., 2024) found that RG1 promotes mesenchymal stem cells (MSCs) to secrete exosomes, which reduce myocardial fibrosis and inflammation by activating the NOTCH signaling pathway to induce macrophage M2 polarization.

**FIGURE 2:**
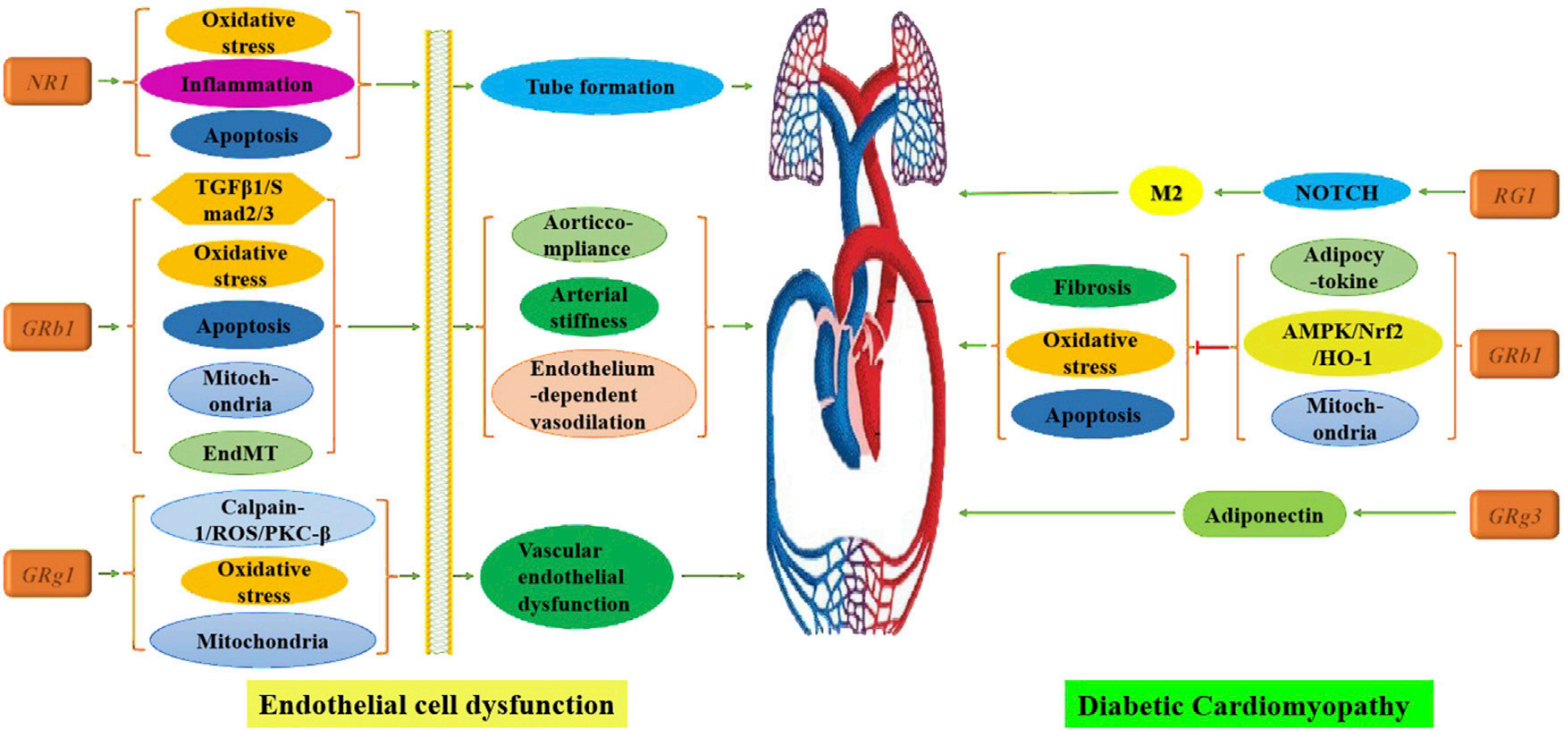
Mechanism of multiple ginsenosides in treating cardiovascular complications of DM.

These findings underscore that ginsenosides not only enhance cardiovascular function by improving endothelial cell function but also restore myocardial function by addressing pathological alterations in cardiomyocytes, as illustrated in Figure 2. Future directions for research include developing a wider range of ginsenosides, investigating their molecular mechanisms, and exploring combined applications with polymer materials.

### 2.2 Digestive system

Diabetes digestive diseases are mainly caused by autonomic neuropathy and microcirculation disorders caused by long-term hyperglycemia, which can involve the gastrointestinal tract, liver and pancreas. We mainly describe different kinds of ginsenosides in the treatment of diabetes and its complications from these three aspects.

#### 2.2.1 Diabetic liver disease

Diabetic patients experience impaired glucose metabolism, disrupting the regulatory balance between the liver and pancreas, which in turn diminishes the liver’s capacity to manage blood glucose levels. Hyperglycemia triggers hepatocyte inflammation through mechanisms such as mitochondrial oxidative stress, endoplasmic reticulum (ER) stress, and reduced lysosomal autophagy, causing substantial hepatocyte damage and subsequent liver function decline (Lange et al., 2022). Rh1 not only inhibits elevations in triglyceride (TG), total cholesterol (TC), and low-density lipoprotein cholesterol (LDL-C) levels but also enhances the secretion of G6Pase and phosphoenolpyruvate carboxykinase (PEPCK) in the gluconeogenesis pathway. Histological analyses further indicate that Rh1 mitigates liver tissue apoptosis and suppresses inflammatory mediators, including NF-κB and NOD-like receptor protein 3 (NLRP3), providing initial evidence of Rh1’s protective role against liver damage in T2DM (Su H. et al., 2022). Additionally, Zhu et al. (2021) reported that Rg5 improves liver injury and hepatocyte apoptosis *via* the insulin receptor substrate-1 (IRS-1)/phosphatidylinositide 3-kinase (PI3K)/protein kinase B (AKT) pathway, alleviates hepatic oxidative stress and inflammation, and promotes mitochondrial biosynthesis in T2DM. Thus, Rg1 and Rg5 show promise as natural interventions for T2DM. Further studies suggest that Rb1 has a strong affinity for 15-PGDH and may enhance hepatic glycogen synthesis through a 15-PGDH-dependent mechanism, offering new insights into Rb1’s positive effects on T2DM (Liang et al., 2023).

Inadequate glycemic control in diabetes increases insulin levels, stimulates fat synthesis, inhibits lipolysis, and impairs the transformation of lipids into lipoproteins, which are essential for lipid transport from the liver. Consequently, these lipids accumulate in hepatocytes, leading to hepatic steatosis. Enhancing lipid metabolism in the liver is, therefore, an essential strategy for addressing diabetic liver disease (Guo et al., 2024). Huang et al. (2022) demonstrated that Red Ginseng significantly reduces fasting blood glucose and TG and TC levels in T2DM rats. Both *in vitro* and *in vivo* studies confirm its efficacy in correcting lipid metabolism disorders and alleviating hepatic steatosis, supporting the potential of red ginseng as a functional food for diabetes management. Panax ginseng (PG-MGR), another natural ginseng product, also shows promising effects by lowering fasting blood glucose (FBG), TG, TC, and LDL-C levels, improving insulin resistance and glucose tolerance, and reducing liver damage by decreasing aspartate aminotransferase (AST) and alanine aminotransferase (ALT) expression through the activation of IRS-1/PI3K/AKT and AMPK signaling pathways (Wang D. S. et al., 2022).

To date, research on diabetic liver complications remains limited, focusing mainly on liver function impairment and lipid metabolism. Future studies should aim to develop a wider variety of ginsenosides, investigate their molecular mechanisms in greater detail, and assess potential hepatotoxic effects.

#### 2.2.2 Pancreatic dysfunction

Pancreatic diabetes, a type of diabetes mellitus caused by pancreatic exocrine diseases, is most frequently associated with chronic pancreatitis. Key pathogenic factors include islet dysfunction, insulin insufficiency, insulin resistance, reduced incretin hormone levels, and disruptions in intestinal flora (Vonderau and Desai, 2022). Immune-mediated islet dysfunction plays a critical role in the development of pancreatic diabetes, where macrophage recruitment and activation lead to the release of numerous inflammatory cells, contributing to pancreatic β-cell dysfunction. Studies indicate that the early recruitment and activation of macrophages exacerbate pancreatic cell damage (Wagner et al., 2022). CK has been shown to dose-dependently reduce M1-type inflammatory cytokine expression in macrophages *via* the PPAR γ/NF-κB signaling pathway, effectively improving insulin resistance and glucose tolerance (Xu et al., 2022). Similarly, Rg1 reduces inflammation and insulin resistance, while also activating AMPK and inhibiting mammalian target of Rapamycin (mTOR)-mediated autophagy and apoptosis (Chen et al., 2023; Zong et al., 2023). Additionally, Rh4 has demonstrated a significant effect in alleviating diabetes symptoms, normalizing glucose metabolism, and enhancing insulin secretion, primarily through increased Nrf2 expression. Elevated pancreatic inflammation levels decrease insulin secretion, but Rh4’s effects include promoting Nrf2 nuclear translocation and boosting insulin production by activating pancreatic and duodenal homeobox-1 (PDX-1) and glucose transporter-2 (GLUT2) signaling pathways. Investigating the Nrf2 pathway offers promising potential as a therapeutic strategy to address pancreatic β-cell dysfunction in diabetes (Liu et al., 2021). Experimental results further indicate that preparations with spherical structures exhibit smaller particle sizes, enhanced penetrative ability, and an encapsulation rate as high as 99.8%, significantly improving fasting insulin (FINS) levels and insulin sensitivity index (ISI) (Han et al., 2024).

Glucagon-like peptide-1 (GLP-1) is a hormone released during the intestinal digestion and absorption of nutrients, which stimulates insulin secretion. However, in cases of pancreatic insufficiency, nutrient absorption is compromised, leading to reduced incretin hormone release and subsequent increases in blood glucose levels. Rk1 has been found to activate the anti-apoptotic effects of the PI3K/AKT/B-cell lymphoma-2 (Bcl-2) signaling pathway by directly targeting and activating the insulin-like growth factor 1 receptor (IGF-1R). Additionally, Rk1 reduces pancreatic weight and increases pancreatic insulin levels, thereby protecting the pancreas from high-fat diet (HFD)-induced diabetes (Vong et al., 2024).

In summary, substantial research has clarified the mechanisms underlying pancreatic diabetes. Future efforts may focus on combining various ginsenosides for diabetes treatment, aiming for complementary effects and exploring multiple drug delivery routes to optimize therapeutic efficacy.

#### 2.2.3 Abnormal intestinal metabolism

L cells, a type of intestinal endocrine cell dispersed throughout the gastrointestinal tract, secrete several critical peptide hormones, including GLP-1, GLP-2, polypeptide YY (PYY), and gastric oxyntic regulator. These hormones play vital roles in promoting insulin secretion, regulating appetite, and managing blood glucose levels and body weight. Recent studies have identified L cells as pivotal targets for diabetes treatment, with CK emerging as the most extensively researched agent. CK has been shown to alleviate ileal epithelial injury and intestinal fibrosis by increasing levels of lithocholic acid (LCA) and deoxycholic acid (DCA). Additionally, CK promotes L-cell transformation and enhances GLP-1 release by upregulating genes associated with L-cell differentiation (Tian et al., 2022a). The underlying mechanisms involve pathways such as the gut microbiota-bile acid (BA)-TGR5 pathway and the RhoA/ROCK/YAP signaling pathway (Tian et al., 2021; Tian et al., 2022a).

The gut microbiota plays a pivotal role in the development of type 2 diabetes, exerting regulatory effects on the body’s metabolic and inflammatory responses. Disturbances in gut microbiota are linked to dysregulation of immune cells and elevated levels of inflammatory cytokines, making them significant contributors to various inflammation-mediated diseases. Rb1 has been shown to reverse intestinal microbiota disorders in diabetic mice by increasing the abundance of *Umbellifera mites* while decreasing the levels of *Aristipes*, *Preethylene Silkworms*, *Stinky bacterium*, and *Anaerobic Proplasma*. Furthermore, Rb1 altered the composition of free fatty acids (FFAs) in fecal metabolites, reducing α-linolenic acid, oleic acid, arachidonic acid, palmitic acid, and stearic acid (Zhou et al., 2023). Rd enhanced the abundance of beneficial bacteria through the activation of the AKT pathway, while simultaneously decreasing the abundance of conditionally pathogenic bacteria (Wang et al., 2023). Rg1 also contributed by increasing the proportion of *Leptospira* and *Clostridium leptoilea* and decreasing *Lactic acid bacteria* (Peng et al., 2022). These findings indicate that ginsenosides may function as potential prebiotics, regulating specific gut microbes and related metabolites that are crucial in diabetes-related metabolic disorders and insulin resistance.

**FIGURE 3:**
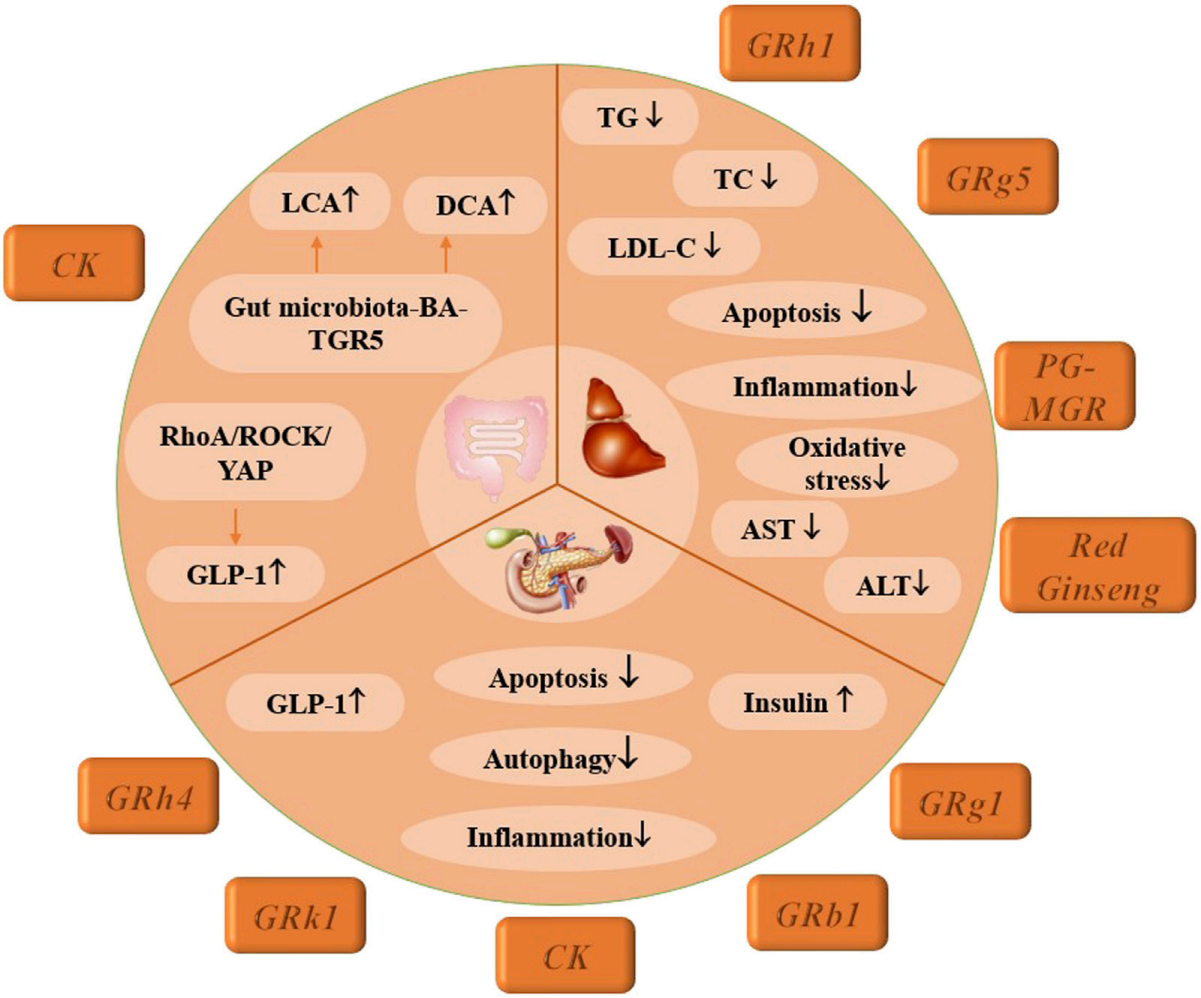
Mechanism of multiple ginsenosides in treating digestive system-complications of DM.

These findings not only elucidate the mechanisms through which CK affects intestinal L cells but also establish a molecular foundation for further exploring CK as a potential therapeutic agent for the treatment of T2DM, as illustrated in Figure 3.

### 2.3 Diabetic nephropathy

DR is one of the most severe complications of diabetes, while DN represents the primary microvascular complication, primarily characterized by diabetic glomerulosclerosis, a glomerular lesion driven by vascular damage.

In its early stages, DN is often asymptomatic, with blood pressure remaining normal or elevated. The incidence of this condition increases with the duration of diabetes. Initially, kidney volume and glomerular filtration rate (GFR) rise, leading to a state of hyperfiltration, followed by the gradual onset of interstitial proteinuria or microalbuminuria (Jung et al., 2021). As the disease progresses, persistent proteinuria, edema, hypertension, and a decrease in GFR can lead to renal insufficiency and uremia, which are significant contributors to diabetes-related mortality.

Research indicates that Fc, CK, and Rg3 can improve urine microalbumin levels in diabetic mice through various mechanisms (Zhou et al., 2020; Chen et al., 2022; Shen et al., 2024). CK mitigates oxidative stress accumulation, decreases levels of pyroptosis-associated proteins, reduces mitochondrial membrane potential collapse, and modulates the expression of mitochondrial fission proteins while increasing mitofusin 2 (Mfn2) expression. CK also remodels the gut microbiota by reducing fungal content and p-prostetones, while increasing lactobacilli levels and decreasing serum concentrations of the histidine-derived microbial metabolite imidazole propionate (IMP). Rg3 treatment activates Toll-like receptor 4 (TLR4), resulting in improved renal histology, significantly reduced apoptosis of renal tubular epithelial cells, and lower fasting blood glucose, creatinine, total cholesterol, and triglyceride levels, as well as reduced expression of inflammatory factors compared to the diabetic group.

Glomerulosclerosis and the hyperperfusion of residual nephron glomeruli in chronic kidney disease are critical factors contributing to further nephron loss. LDL can induce increased apoptosis of mesangial glomerular cells, exacerbating kidney tissue damage. Therefore, alleviating apoptosis is a crucial strategy for treating DN. Chen et al. (2024) reported that Rg3 targets miR-216a-5p, activates the MAPK pathway, inhibits apoptosis, and alleviates kidney damage in diabetic mice. Similarly, Su et al. (2021) found that Rh1 yields comparable effects, with molecular mechanism studies demonstrating that its benefits are linked to apoptosis inhibition *via* the AMPK/PI3K/AKT signaling pathway. Additionally, He et al. (2022) identified that Rb1 significantly reduces diabetes-induced glomerular injury, podocyte apoptosis, and mitochondrial damage—such as glomerular hypertrophy and mesangial stromal dilation—while decreasing the expression of apoptotic proteins.

Additionally, research demonstrates that ginsenosides exert significant effects primarily by reducing oxidative stress, inflammation, and pathological changes in renal histology, with Rg series ginsenosides being particularly notable. For instance, cell experiments have shown that Rg1 effectively lowers urine protein, serum creatinine, urea nitrogen, blood lipid levels, and renal lipid volume in T2DM mice. Pathological analyses indicate that Rg1 treatment alleviates renal damage and glomerular fibrosis (Han et al., 2023; Ji et al., 2023). This therapeutic effect is mediated through the transient receptor potential cation channel 6 (TRPC6)/nuclear factor of activated T Cells (NFAT2) and NADPH oxidase 4 (NOX4)-MAPK signaling pathways.

**FIGURE 4:**
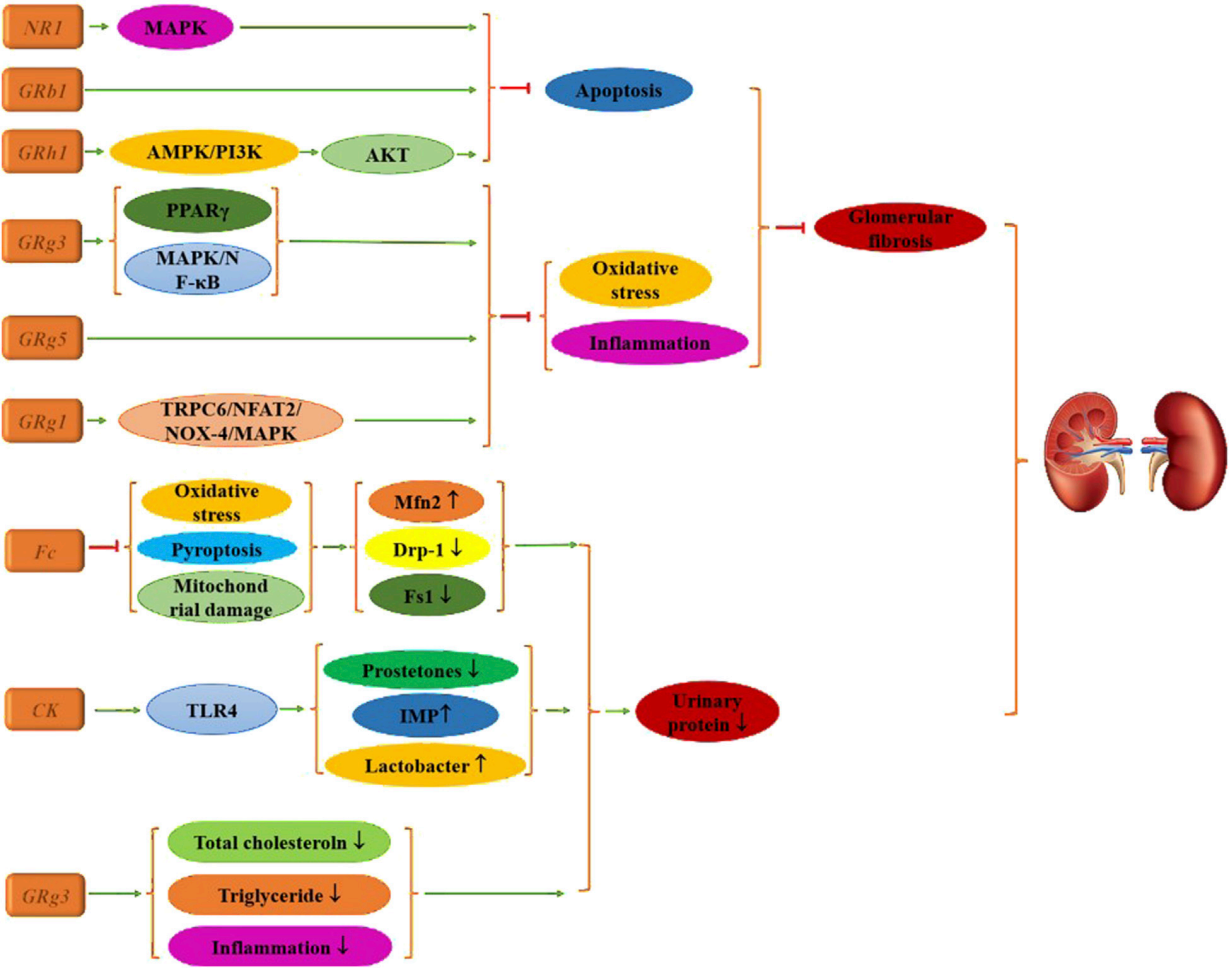
Mechanism of multiple ginsenosides in the treatment of diabetic renal complications.

Rg3 also contributes positively by upregulating PPARγ activity, thereby reducing inflammatory and fibrosis biomarkers (Sui et al., 2023). Concurrently, it enhances insulin (INS) levels, improves blood lipid profiles, mitigates oxidative stress, and restores renal function *via* MAPK and NF-κB signaling pathways (Li et al., 2021), leading to improved renal histopathological outcomes. Rg5 further protects against kidney injury in diabetic mice by inhibiting oxidative stress and the activation of the NLRP3 inflammasome, suggesting its potential as a compound for preventing or managing diabetic kidney injury (Zhu et al., 2020), as illustrated in Figure 4.

DN is a chronic, progressive condition where clinical symptoms often manifest late, and once persistent proteinuria occurs, renal function deteriorates irreversibly and progressively. Currently, most studies focus on a limited number of common ginsenosides. Future research should explore the therapeutic effects of ginsenosides from other varieties, aiming to enhance their impact on kidney lesions and experimenting with various drug delivery methods to improve therapeutic efficacy.

### 2.4 Diabetic neuropathy

Diabetic neuropathy is one of the most prevalent chronic complications of diabetes, impacting both central and peripheral nerves, with distal sensory neuropathy being the most common, accounting for over 50% of all diabetic neuropathies (Mittal et al., 2025).

**FIGURE 5:**
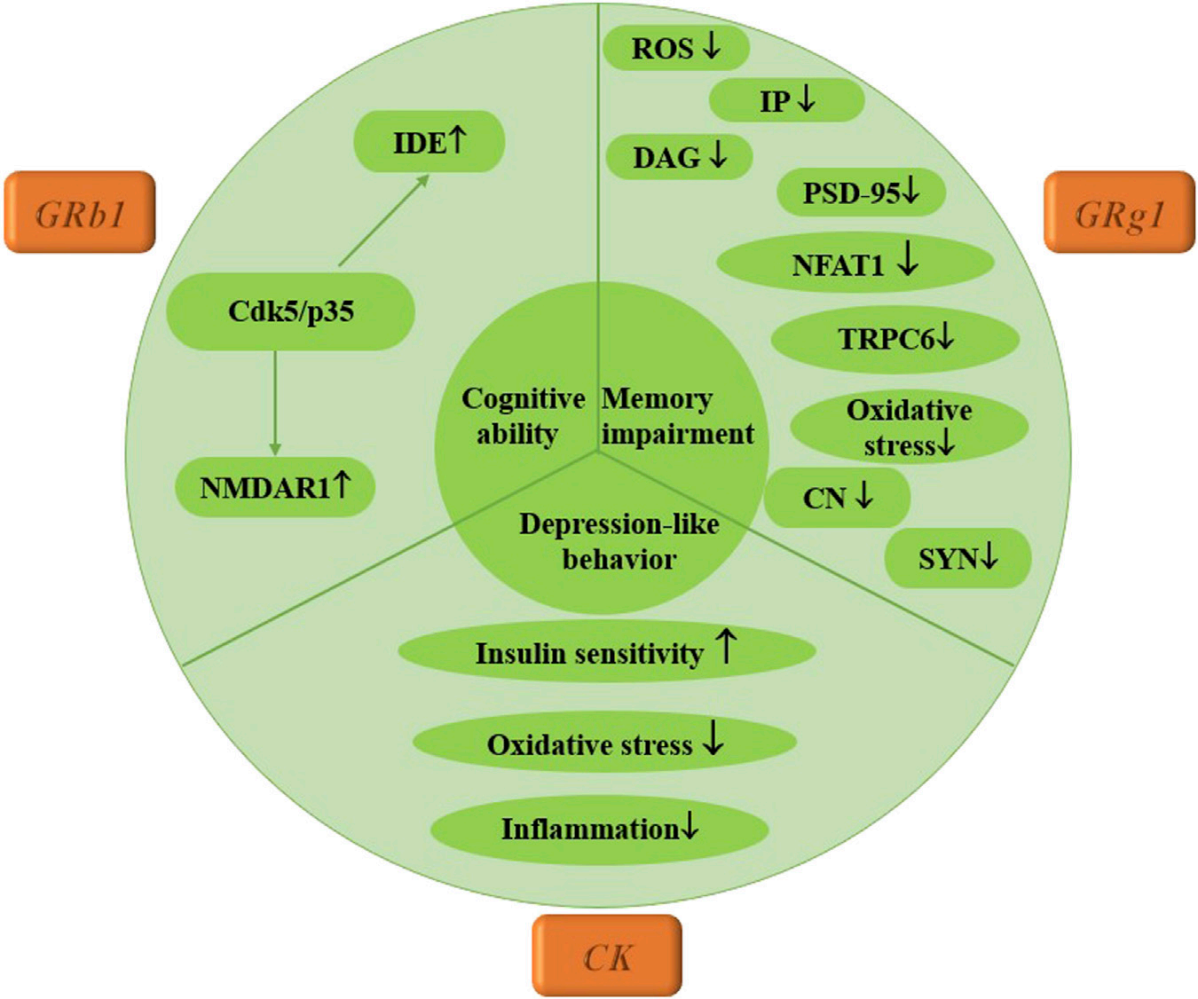
Mechanism of multiple ginsenosides in the treatment of diabetic nervous system complications.

Currently, there is a lack of published studies on the therapeutic effects of ginsenosides specifically for diabetic peripheral neuropathy. This study aims to explore the effects of three ginsenosides on diabetic central neuropathy through assessments of memory impairment, depression-like behaviors, and cognitive abilities in mice. Dong et al. (2023) demonstrated that Rg1 reduces levels of ROS, inositol triphosphate (IP3), and diacylglycerol (DAG), effectively reversing Ca^2+^ overload. This is achieved by downregulating the expression of p-PLC, TRPC6, and NFAT1 nuclear translocation, which alleviates amyloid-beta (Aβ) deposition and enhances postsynaptic density-95 (PSD-95) expression in T2DM mice. Additionally, Li et al. (2020) reported that CK treatment significantly improved behavioral impairments in mice, as CK not only lowered fasting blood glucose levels but also enhanced lipid metabolism, glucose tolerance, insulin sensitivity, and dyslipidemia. It further reduced oxidative stress and inhibited inflammatory responses in the hippocampus, alleviating ER stress and suppressing the NLRP3 inflammasome pathway. In addressing cognitive decline associated with diabetes, a 2020 study found that Rb1 improved memory and cognitive function in mice with streptozotocin (STZ)-induced damage. Rb1 also mitigated STZ-induced glucose intolerance by enhancing insulin sensitivity, with these beneficial effects attributed to the inhibition of Cdk5/p35 activity and the upregulation of N-methyl-D-aspartate receptor-1 (NMDAR1) expression in the hippocampus. This research is crucial for understanding the mechanisms by which ginsenoside Rb1 enhances cognitive performance and its implications for the relationship between diabetes and Alzheimer’s disease (AD) (Yang et al., 2020). Furthermore, some researchers have combined Rb1 with small alkali compounds to treat diabetic neuropathy, finding that this combination improved glucose metabolism and insulin resistance and alleviated depression-like behaviors associated with chronic unpredictable stress. Hematoxylin-eosin (HE) and Nissl staining in animal experiments indicated that neurons were protected from pathological and morphological changes. Thus, the combination of small alkali and Rb1 may hold clinical value for treating patients with diabetes and co-occurring depression (Zhang J. H. et al., 2021). Currently, scholarly attention has primarily focused on diabetic central neuropathy, while the therapeutic mechanisms of ginsenosides are extensive. Future research should delve deeper into the role of ginsenosides in the treatment of diabetic peripheral neuropathy, providing a robust theoretical foundation for their clinical application, as illustrated in Figure 5.

### 2.5 Diabetic retinopathy

DR is one of the most prevalent microvascular complications of diabetes, resulting from chronic diabetes mellitus and leading to various fundus lesions, including microangiopathy, hard exudates, cotton wool spots, neovascularization, vitreous hyperplasia, macular edema, and even retinal detachment (Bryl et al., 2022).

**FIGURE 6:**
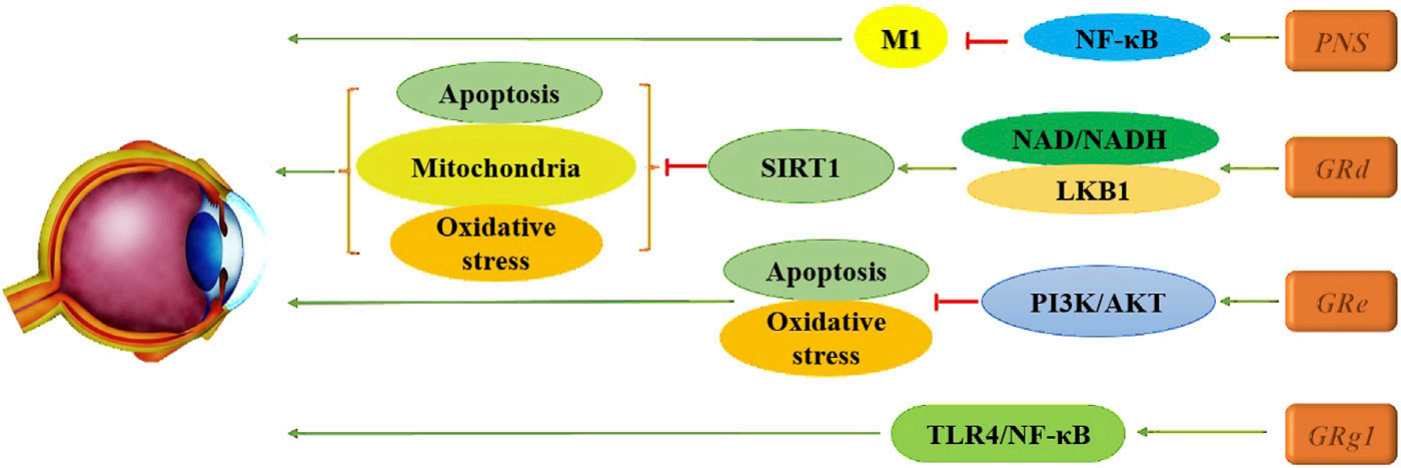
Mechanism of multiple ginsenosides in treatment of DR.

Wang et al. (2024a) found that Panax notoginseng saponins (PNS) significantly increased retinal core layer thickness and mitigated the rise in retinal cell-free capillaries while markedly reducing microglial activation. Furthermore, PNS inhibits the activation of the NF-κB signaling pathway in M1 cells and suppresses cellular inflammatory responses, thereby alleviating DR and reducing retinal inflammation. Xue et al. (2023) also identified considerable efficacy of Rg1, demonstrating its ability to effectively lower levels of intracellular inflammatory cytokines and growth factors. This suggests a potential therapeutic strategy for DR through the upregulation of miR-216a-5p and the inhibition of the TLR4/NF-κB signaling pathway. Additionally, the PI3K/AKT pathway has been explored, with evidence that Re can counteract high glucose-induced (HG) decreases in RF/6A cell viability, reduce the apoptosis rate, and inhibit oxidation-related enzymes. This action leads to decreased ROS production and mitigates HG-triggered damage to RF/6A cells, providing cytoprotective effects associated with the activation of the PI3K/Akt pathway (Xie et al., 2020). Notably, the use of LY294002, a PI3K inhibitor, partially reversed the effects of Re on apoptosis-related proteins, indicating that Re may improve HG-induced retinal angiogenesis. Diabetes mellitus typically elevates retinal oxidative stress levels, generating large amounts of ROS *via* pathways such as advanced glycation end products (AGE), the polyol pathway, the hexosamine pathway, and the PKC pathway. This oxidative stress damages the retina, leading to further oxidative damage and apoptosis, creating a vicious cycle by activating additional cytokines that upregulate oxidative stress levels. In this context, Tang et al. (Tang et al., 2022) demonstrated through various *in vitro* and *in vivo* experiments that Rd enhances the interaction between AMPK and silent information regulator family protein 1 (SIRT1) by increasing nicotinamide adenine dinucleotide (NAD)/NADH levels and facilitating liver kinase B1 (LKB1) deacetylation in endothelial cells. This mechanism effectively reverses hyperglucose-induced activation of NADPH oxidase 2 (NOX2), oxidative stress, mitochondrial dysfunction, and endothelial cell apoptosis. These findings support the clinical development of Rd as a pharmacological intervention, presenting it as a novel potential vasoprotective agent for early DR. In summary, ginsenosides have shown significant promise in the treatment of DR, highlighting their potential for clinical application in the management of this complication, as illustrated in Figure 6.

### 2.6 Other complications

In addition to the previously mentioned systemic and organ complications, diabetes can lead to various other complications, which require systematical discussion due to a lack of comprehensive literature.

Tendinopathy, characterized by muscle cell apoptosis and damage to the extracellular matrix, can be influenced by diabetic conditions. CK has demonstrated efficacy in counteracting high glucose-induced apoptosis, inflammation, and oxidative stress in cultured cells. Specifically, CK normalizes the expression of matrix metalloproteinases-9 (MMP-9), MMP-13, and tissue inhibitor of metalloproteinase-1 (TIMP-1), while enhancing the expression of PPARγ and antioxidant enzymes (Cho et al., 2024). This results in improved mitochondrial membrane potential, increased glucose uptake and glycogen synthesis, and enhanced mitochondrial mass (Li et al., 2023), highlighting CK’s therapeutic potential in hyperglycemic tendinopathy. Additionally, in an HFD and HG diabetic mouse model, Rg3 treatment significantly reduced triglyceride and glucose levels in C2C12 myoblasts. It promoted myoblast differentiation, inhibited mitochondrial dysfunction, increased climbing distances, and mitigated muscle atrophy. These beneficial effects are associated with the phosphorylation of AMPK and Forkhead Box O3 (FoxO3) and the inhibition of Smad3 phosphorylation (Wang M. et al., 2024).

Currently, only one study published in 2022 has investigated the role of ginsenosides in treating diabetic lung tissue injury, utilizing Rb1 (Su H. et al., 2022). The findings revealed that Rb1 treatment not only significantly reduced the apoptosis rate of lung tissue cells—2.23 times in the diabetic group compared to 1.73 times in the treatment group—but also decreased oxidative damage and inflammatory infiltration in the lungs. This was achieved by lowering the expression of various inflammatory factors, including interleukin-6 (IL-6), interleukin-1α (IL-1α), and tumor necrosis factor-α (TNF-α).

Poor wound healing is a prevalent chronic complication of diabetes, influenced by factors such as abnormal inflammation, reduced granulation tissue content, impaired angiogenesis at the wound site, and peripheral neuropathy. Rg1 has been shown to enhance the proliferation, migration, and angiogenesis of human umbilical vein endothelial cells (HUVECs) while reducing apoptosis (Huang et al., 2021). Additionally, Rg5 inhibits the expression and activity of SLC7A11 through physical binding, alleviating the negative regulation of anaerobic glycolysis and promoting erythropoiesis of dendritic cells (Xia et al., 2023). Rg1’s effects are mediated by the downregulation of the key nucleic acid miR-489-3p and the activation of the PI3K/AKT/eNOS signaling pathway. Consequently, ginsenosides have potential as adjuvant therapeutic agents to support wound healing in patients, particularly those with diabetic foot ulcers.

Moreover, two clinical studies have explored the effects of ginsenosides. One study found that the addition of ginseng to drug treatment in diabetic patients improved central systolic blood pressure and pulse wave formation, without directly affecting endothelial function (Jovanovski et al., 2020). In another study involving Rg3-KR intervention, a reduction in HbA1c levels was observed (−0.35% ± 0.1% [-3.8 ± 1.1 mmol/mol], p = 0.02) at 12 weeks, with no adverse safety outcomes reported. These findings suggest that ginsenosides may offer clinical benefits when incorporated into polypharmacy and lifestyle interventions for diabetes management (Jovanovski et al., 2021).

## 3 Prospects of ginsenosides in the treatment of type 2 diabetes and its complications

To date, numerous studies have demonstrated the significant potential of ginsenosides in the treatment of type 2 diabetes, elucidating various related mechanisms to some extent. However, the application of ginsenosides as a therapeutic strategy remains limited. Key challenges include: 1) Most research has focused on a single variety of ginsenosides in cell or animal experiments, with limited exploration of polypharmacy; 2) The predominant route of administration in most studies has been oral, necessitating further investigation of multiple delivery routes to enhance drug utilization; 3) There is a need for in-depth studies on the specific concentrations of ginsenosides in bone and their potential toxic side effects in other organs; and 4) With the emergence of numerous new biochemical materials in recent years, there is an opportunity to explore the synergistic effects of ginsenosides in combination therapies.

## 4 Conclusion

As research into ginsenosides for diabetes treatment expands, this review outlines the possible mechanisms by which different types of ginsenosides exert therapeutic effects, summarizing their specific signaling pathways and key factor mechanisms. Nevertheless, this therapeutic approach is still in the early stages of clinical translation, facing numerous obstacles to clinical application, such as the need for combination drugs, optimization of routes of administration, bioavailability, and integration with biochemical materials. With the growing recognition of ginsenosides’ roles in diabetes management, they hold considerable promise as a new therapeutic agent for diabetes and its associated complications, positioning them as strong candidates for future drug development.
